# Congenital Suipoxvirus Infection in Newborn Piglets in an Austrian Piglet-Producing Farm

**DOI:** 10.3390/microorganisms12091757

**Published:** 2024-08-24

**Authors:** Lukas Schwarz, René Brunthaler, Angelika Auer, René Renzhammer, Ursula Friedmann, Andrea Buzanich-Ladinig

**Affiliations:** 1 Clinical Centre for Population Medicine in Fish, Swine and Poultry, Clinical Department for Farm Animals and Food System Science, University of Veterinary Medicine Vienna, 1210 Vienna, Austria; rene.renzhammer@vetmeduni.ac.at (R.R.); andrea.ladinig@vetmeduni.ac.at (A.B.-L.); 2 Centre for Pathobiology, Department of Biological Sciences and Pathobiology, University of Veterinary Medicine Vienna, 1210 Vienna, Austria; rene.brunthaler@vetmeduni.ac.at (R.B.); angelika.auer@vetmeduni.ac.at (A.A.); 3 PFI DR VET—Die Tierärzte OG, 8403 Lang, Austria; ursula.friedmann@dr-vet.at

**Keywords:** pox-like skin lesions, hydropic degeneration, intracytoplasmic inclusion bodies

## Abstract

In February 2020, a fourth parity sow gave birth to a litter of piglets with four piglets presenting pox-like skin lesions. Lesions were distributed over the whole skin surface and ulcerative lesions were also observed on the mucosa of the oral cavity. The skin lesions were described as looking like pox lesions. Virological and histopathological investigations confirmed congenital suipoxvirus infection. Since there is no effective treatment available, the farmer was recommended to improve hygiene. No further cases occurred after this single event. In the past, suipoxvirus infections were mainly related to improper hygiene conditions and to pig lice as vectors. Today, conventional pigs are usually kept under good hygienic conditions and pig lice are not reported anymore to occur in Austrian conventional pig farming systems. Therefore, we speculate, that other living vectors, such as the stable fly, may play a role in the transmission of suipoxvirus between and within farms and in the occurrence of congenital suipoxvirus infections in neonatal piglets.

## 1. Introduction

Swinepox (SP) is a viral disease sporadically occurring in swine [1] all over the world [2,3,4,5,6,7,8,9]. It was first described in Europe in 1842 [10] and in North America in 1929 [11]. All age groups of swine can become infected. Transmission of SP virus (SPV) mainly occurs via living vectors such as biting insects [1,12,13]. Horizontal transmission may also happen via contact with nasal or oral secretions. In rare cases, vertical transmission leads to congenital SP infections resulting in more- or less-severe clinical outcomes [2,3,7,14]. SPV enters the body of its host via skin lesions. The virus replicates in the cytoplasm of keratinocytes of the stratum spinosum, leading to hydropic degeneration [15,16]. In experimental infections, the virus can be isolated from skin lesions three days after infection [17]. The dissemination of the virus from the primary infection site to other organs still remains unclear, though congenital infection of foetuses is hypothesized to be related to viremia in sows. However, to date, the detection of SPV in blood or serum of infected animals has been unsuccessful [3,7,17].

The clinical outcome of SPV infection is age-dependent, with increasing severity in younger animals. In adults, clinical signs are usually mild and the infection is self-limiting [1]. On the other hand, congenital SPV infections may end up in stillborn piglets or may cause increased mortality during the first days of life [1,7]. Lesions of SPV infections are described as multifocal, eruptive dermatitis, with skin lesions commonly observed on the abdomen, the inner surface of the legs and ears and, in some cases, on the face [18,19], developing from maculae to papules and from vesicles to umbilicated lesions with pustular content, which encrust and heal afterwards [11,17,20]. While the presumptive diagnosis of SP is based on the occurrence of characteristic pox lesions on the skin, differential diagnoses such as other vesicular diseases of pigs, skin diseases associated with classical swine fever, erysipelas, pityriasis rosea, bacterial dermatitis, sunburn, parasitic skin diseases and nutritional disorders should be considered [1]. A final diagnosis is achieved by observation of typical histopathologic lesions in combination with PCR-based detection of virus DNA. There is no specific treatment available. In the case of secondary bacterial infections, antimicrobial treatment is indicated. Preventive control measures include optimized and good animal husbandry, including ectoparasite control [1].

The purpose of this case is to describe the occurrence of congenital swinepox virus infection in newborn piglets of a single litter in a conventional piglet-producing farm in Austria.

## 2. Case Presentation

The present case occurred in a family-owned piglet-producing farm in Styria, Austria. This farm produced conventional piglets with 146 sows (local Large White) in a three-week batch-farrowing interval and with a suckling period of 28 days. Piglets were nursed on site until they were sold with an average body weight of 30 kg. Semen for artificial insemination was purchased from the local official boar stud. The replacement rate was about 38%. One boar was used as a teaser boar. Animals were fed a corn silage-based diet added to dry feed which was given by hand to the sows. Gilts were purchased from one gilt-producing farm. No isolation unit was available on the case farm and gilts were introduced directly into the sow herd. Sows were regularly vaccinated against Porcine Reproductive and Respiratory Syndrome with a modified live-virus PRRSV-1 vaccine every four months, against *Escherichia coli*- and *Clostridium perfringens*-related diarrhoea in suckling piglets with a combined vaccine prior to farrowing, and against parvovirosis, leptospirosis and erysipelas also with a combined vaccine three weeks postpartum. Anthelminthic treatment was performed every four months with alternating ivermectin and fenbendazol.

In July 2019, the herd struggled with an outbreak of pandemic H1N1 influenza A virus (panH1N1). This led to the decision of the herd veterinarian to introduce a vaccination against panH1N1 using a commercially available vaccine. The panH1N1 outbreak resulted in the abortion of four sows and in anorexia in the acute phase of the outbreak.

In February 2020, the University Clinic for Swine became involved in a case of pox-like skin lesions in neonatal suckling piglets. The herd veterinarian had never seen such lesions before on any of his farms. Affected suckling piglets were born by a sow of fourth parity. This sow gave birth to 16 live-born piglets and one dead-born without any skin lesions. Four of the live-born piglets presented with pox-like skin lesions distributed over the whole skin surface of the body. One out of the four affected piglets died on the third day of life. The sow and the remaining littermates did not show any signs of skin lesions. The remaining 19 litters of that batch were also clinically unsuspicious.

### Diagnostic Work-Up

**Figure 1:**
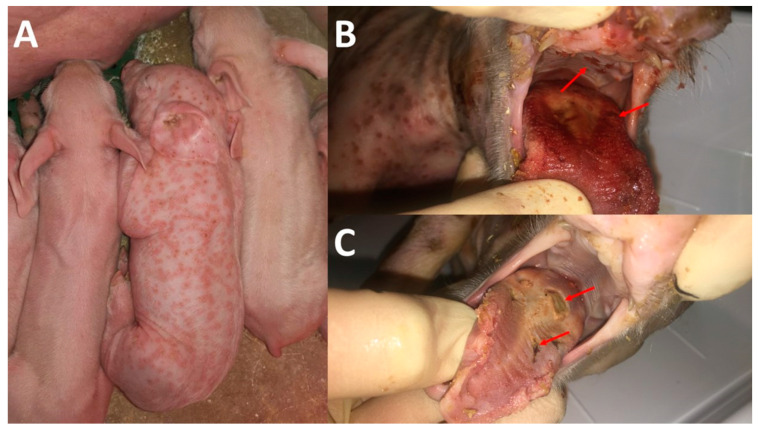
Severely affected neonatal piglet presenting with characteristic pox-like skin lesions. This piglet did not suckle, while the littermates did (**A**). Two piglets with pox-like lesions on all the skin of the body, showing additionally ulcerative lesions in the mucosa of the oral cavity. The red arrows indicate affected parts of the oral mucosa (**B**,**C**).

In February 2020, the herd veterinarian conducted a farm visit. All different compartments were visited and checked. The focus was on the farrowing unit, as animals were unsuspicious in all other compartments. According to the herd veterinarian, only one out of 20 litters was affected; in total, four piglets showed the described skin lesions. One piglet that died on the third day of life showed severe lesions and was stored for further diagnostics in the refrigerator. One affected piglet presented only a few pox-like lesions on the skin, but no further clinical abnormality could be found. The two remaining affected suckling piglets had a moderately reduced general behaviour and stopped suckling immediately after they took the nipples of the sow into their mouth (Figure 1).

**Figure 2:**
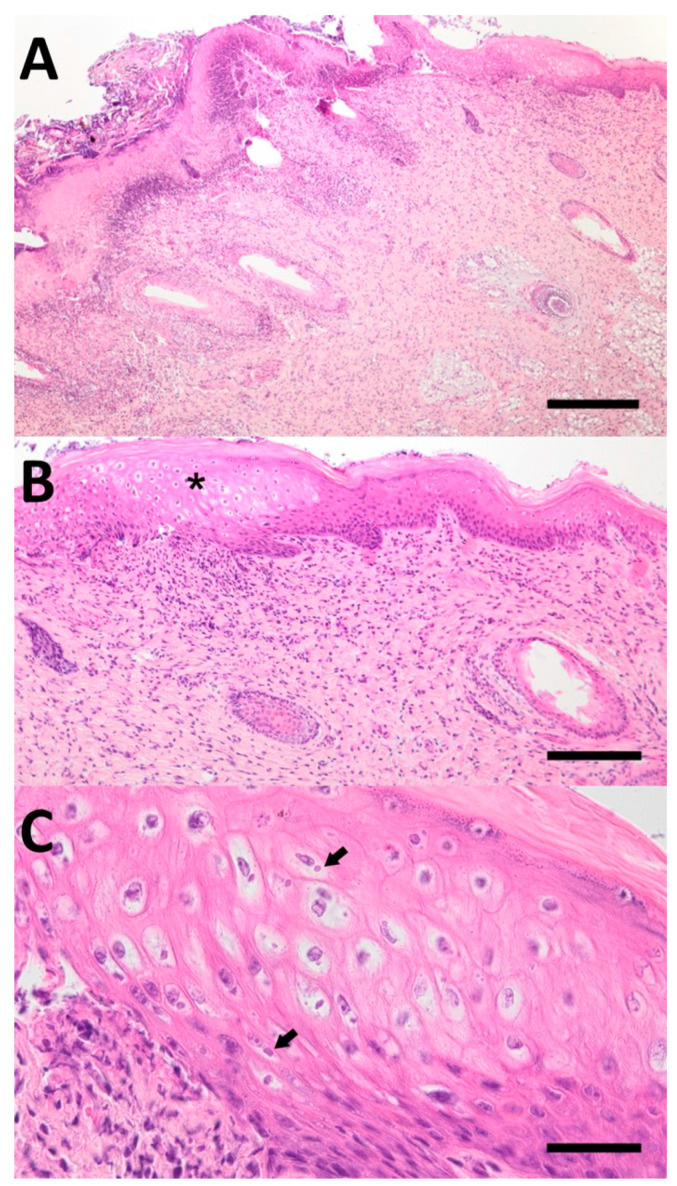
(**A**) High-grade necrosis of keratinocytes with superficial crusts, intralesional bacteria and plant material. The underlying dermis is infiltrated by numerous neutrophilic granulocytes. Hydropic degeneration of the epidermis can be seen in the marginal area of this lesion; hematoxylin and eosin; bar = 400 µm. (**B**) Zone of marked hydropic degeneration of keratinocytes with concomitant epidermal hyperplasia (asterisk); hematoxylin and eosin; bar = 160 µm. (**C**) Hydropic degeneration of the stratum spinosum keratinocytes with eosinophilic intracytoplasmic inclusion bodies (arrow); hematoxylin and eosin; bar = 40 µm.

A clinical examination showed pox-like lesions and severe lesions of the mucosa of the oral cavity, including the tongue (Figure 2). No further clinical abnormalities were found. The two severely affected suckling piglets were euthanized for animal welfare reasons, due to starvation, and for further diagnostics.

No investigations were performed prior to the farm visit. The affected piglet, which died on the third day of life, and the two freshly euthanized piglets were used for a further diagnostic work-up at the University of Veterinary Medicine, Vienna. Tissue samples fixed in formalin from altered skin regions, lungs, heart, lymph nodes, liver, kidney, and intestine were forwarded to the Institute of Pathology at the University of Veterinary Medicine Vienna for histopathologic examination, as described by Kang and colleagues [14]. Tissue samples of characteristic skin lesions were investigated for poxvirus DNA by PCR at the Institute of Virology of the University of Veterinary Medicine Vienna [21]. Additionally, pooled organ tissue samples (kidney, spleen, lymph node and tonsil) from each piglet were sent to the Austrian Agency for Health and Food Safety, AGES IVET Mödling, Austria, to be included in the national surveillance program in order to exclude African and classical swine fever.

In the case of pox-like skin lesions, the list of differential diagnoses includes several diseases, as mentioned before [1]. However, since Austria has been free of notifiable vesicular diseases in pigs since 1981, and since a different, more severe, clinical outcome for age groups other than neonatal piglets could be expected in the case of vesicular diseases, it was decided not to include the vesicular diseases into the list of differentials, primarily. Additionally, Seneca Valley virus had not been described in Europe at the time the case occurred, and was therefore evaluated as implausible in terms of differential. Also, for the other non-infectious and parasitic differential diagnoses, it was assumed that they were of low relevance, since none of them was described as occurring in neonatal piglets. Therefore, only swinepox, bacterial dermatitis and African and classical swine fever were included in the diagnostic work-up.

## 3. Results

Gross pathology revealed disseminated characteristic roundish-to-oval swinepox lesions on the outer skin of the entire carcass with typical erythematous macules, papules, and vesicles with superficial serocellular crusty coatings. All three piglets also showed oligofocal, partly high-grade ulcerations of the tongue. Histopathologic examination of the altered skin areas demonstrated ulcerations with superficial crusts, as well as numerous intralesional bacteria and plant material. In the marginal zones of those lesions, severe epidermal hyperplasia with marked hydropic degeneration of the stratum spinosum keratinocytes was observed. In addition, several eosinophilic intracytoplasmic inclusion bodies could be detected within the infected cells. The dermis showed noticeable infiltration with neutrophilic granulocytes. Furthermore, two of the three animals presented a low-grade purulent bronchopneumonia. No significant pathomorphological changes could be detected in the remaining organ samples.

For virological testing, skin samples were homogenized and nucleic acids were extracted by using the QIAamp Viral RNA Mini QIAcube Kit (Qiagen, Hilden, Germany). Despite its name, this kit allows extraction of RNA, as well as DNA, from different sample materials. PCR, according to Li et al. (2010), using the primer pair for detection of low-GC orthopoxviruses was performed and gave positive results for the samples of all three piglets [21]. A Sanger-sequenced PCR product (168 bp) was compared to sequences from Genbank (NCBI, https://blast.ncbi.nlm.nih.gov/Blast.cgi, last accessed 25 March 2020) and showed 100% concordance with published swinepox sequences.

Due to all performed investigations and due to the obtained results, the final diagnosis for the affected piglets was congenital swinepox infection.

### Treatment and Further Outcome of the Case

Since no treatment is available and since swinepox is of relatively low economic relevance, prevention concerning good animal husbandry conditions and good hygiene, including insect and ectoparasite control, is mandatory. In the case of secondary bacterial infections (e.g., caused by *Staphylococcus hyicus*) antimicrobial treatment may be indicated.

The herd veterinarian reported that three additional piglets of the affected litter developed pox-like lesions before weaning; these were self-limiting and healed over time, as did the lesions from the fourth piglet affected by congenital pox lesions. No specific treatment was implemented and no further infections in any of the other pigs occurred. After more than one year, the herd veterinarian reported that the case of congenital swinepox infection was obviously a single event which did not recur repeatedly, either in the sow of the affected litter or in any other pigs.

## 4. Discussion

In this case, herd congenital SPinfection was diagnosed based on the detection of virus DNA using PCR and based on pathognomonic histopathologic findings, such as epidermal hyperplasia, hydropic degeneration of the keratinocytes of the stratum spinosum and intracytoplasmic inclusion bodies of the infected cells. Similar to the known literature, not all newborn piglets were affected, as just four animals out of sixteen presented with typical pox-like lesions after birth [2,22]. In this herd, SP had never been diagnosed before, either in adult pigs or in neonatal piglets. As congenital SP infections are rarely observed and diagnosed, there is only little knowledge available on the pathological mechanisms [3,7,15,22,23]. However, it is not yet fully understood how SPV passes the placental barrier and infects the foetuses [1]. As congenital SP infections seldom occur and no data at least are available on the prevalence of the SP virus in Austria, it can just be speculated as to whether the virus was already prevalent on that farm or if it was introduced, e.g., via the purchased gilts or other sources such as biting insects. Since the gilts were introduced into the sow herd without any isolation and adaption phase, one may speculate that virus was introduced by gilts. However, a new introduction of SPV into a naïve herd most probably should have resulted in clinically visible pox lesions in several age categories of swine on that farm, with a focus on the younger animals [3], as was recently reported in Sicily [24]. The herd veterinarian of the gilt-producing farm was contacted, and reported that SPV infections had never been observed in the gilt-producing farm. Therefore, it seems to be more realistic to state that SPV was already prevalent on that farm and that the fourth-parity sow either never had contact with SPV or its immunity was suppressed by a still unknown immunosuppressive event. One may speculate, that this immunosuppression of the dam was caused by an infection with the pandemic H1N1 influenza virus during the outbreak in August 2019. As H1N1 infections in pigs cause an acute inflammatory response in the lungs and increased counts of immunosuppressive Tregs, this may have altered the systemic immune system of the pigs also in the case herd [25]. In general, it seems that the real prevalence of SP is underestimated, due to the fact that the infection with SPV in non-congenitally infected animals results in mild clinical signs, which are often neglected by the farmers [26]. However, under certain circumstances, e.g., bad sanitary and hygiene conditions, SP may result in a herd prevalence of nearly 20% [24].

Live vectors may be another possibility for the introduction of the virus into the case herd. The farm was located in a part of Styria, which is one of the high-pig-dense areas in Austria [27]. As nowadays pig lice do not really play a role in conventional pig production anymore, one may speculate that other blood-sucking insects, such as *Stomoxys calcitrans* or tabanids may have overtaken this niche. *Stomoxys calcitrans* is known to fly over a distance of more than one kilometre [28]. The topographic structure of the location of the case farm would make it possible at least for stable flies to transmit SPV from one farm to another. The case farm is surrounded by several small backyard farms within a three-kilometre distance. Furthermore, Austria is a country with quite a high population density of wild boars. As the case herd was located in a region with several connected forest areas, it may be assumed that wild boars are living quite close to the case herd. The local hunter responsible for the hunting ground surrounding the case herd stated that in the hunting period of 2019/2020 a wild boar herd was observed. Furthermore, he stated that the corresponding hunting ground is a migration corridor for wild boars from the north-eastern to the southern and western forest areas. The possibility that SPV may have been introduced to the herd via blood-feeding insects that fed on wild boars prior to the entry into the case herd cannot be excluded. The latter statement is very speculative, as hardly any published data are available in Austria on the prevalence of SPV in the domestic pig and wild boar population [6].

However, as there is no specific treatment available, except for secondary bacterial infections of the skin lesions, SP infections in herds are usually self-limiting [1]. Epidemiologically, pig lice and other biting insects, such as the stable fly, play an important role in the transmission of SPV between pigs [1,29]. Disease control is achieved by a strict hygiene program and a thorough control of the louse population [29]. The farmer did not change any procedural steps in recent years and pig louse infestation had never been recognized, similar to the other reported cases [7,24]. The level of hygiene and biosecurity of the case herd may improve via implementation of a strict cleaning and disinfection program. Moreover, for restocking sows, at least a separated isolation unit should be introduced, to reduce the risk of direct introduction of infectious agents into the breeding stock and to offer an adaptation period to the restocking sows for developing a robust immune response either after natural exposure or after vaccination.

Whether a congenital or non-congenital SPV infection needs any intervention, has to be critically evaluated. The farmer and herd veterinarian of the case herd did not change anything once the final diagnosis was achieved. No special insect control was implemented, nor was any special adaption of the cleaning and disinfection procedure carried out. Since there are no effective veterinary medical products available, either immune-prophylactically or on a therapeutic level, SPV infections may just need therapeutic and prophylactic measures in cases of secondary infections, such as bacterial infections of pox lesions [1,6].

This case supports the fact that congenital SPV infections are usually of relatively low morbidity, with high case fatality for affected suckling piglets, and of a short duration [1,22]. However, in the case of congenital SPV infections resulting in mucosal lesions in the oral cavity, it has to be carefully evaluated by farmers and herd veterinarians whether affected suckling piglets are able to suckle or if they stop suckling due to these very probably painful lesions. In the latter case, euthanasia may be indicated, due to animal welfare reasons, at an early time point. To control SP infections in a farm, implementation of professional hygiene and insect control measures, which may also include stable flies as possible vectors, should be carried out to decrease any risk of transmission within a farm and between animals.

## Data Availability

All data generated or analysed during this case report are included in this published article.
